# Three Bacterial Endophytes Enhanced Plant Growth and Yield and Reduced the Severity of *Phytophthora capsici* in Bell Pepper and Tomato Plants in the Field

**DOI:** 10.3390/plants15091301

**Published:** 2026-04-23

**Authors:** Daniel Ambachew, Margaret T. Mmbaga, Richard Hall, Peter Eyegheleme, Mustapha Olawuni, Jamille Robinson, Emily Rotich

**Affiliations:** 1 Department of Agriculture and Engineering, Tennessee State University, Nashville, TN 37209, USA; ethiodan@gmail.com (D.A.); bladesandowls@gmail.com (R.H.); petereyegheleme@gmail.com (P.E.); moluwanni@tnstate.edu (M.O.); jrobinson84@tnstate.edu (J.R.); 2 Corteva AgriScience, Indianapolis, IN 46268, USA; 3 Biomedical Science, South College, Nashville, TN 37214, USA; erotich@south.edu

**Keywords:** antagonistic association, mutualistic association, plant defense, crop yield, disease management, biological fungicides

## Abstract

Naturally abundant endophytes colonize plants internally without causing harm to their host plants. Endophytes are likely to occupy the same ecological niches as phytopathogens and thus have a high potential to be effective biological control agents. Their demonstrated ability to suppress more than one plant pathogen suggests that they can offer a viable alternative to chemical fungicides and a strategy for decreasing the inoculum potential of soil-borne pathogens. Some biocontrol endophytes are also known to improve soil health and the overall health of plants. However, the results in greenhouse studies do not always translate to consistent field efficacy. In this study, the efficacy of three endophytic bacterial isolates (PRT (*Bacillus subtilis*), PSL (*Bacillus amyloliquefaciens*), and IMC8 (*Bacillus thuringiesis*) were evaluated against *Phytophthora capsici* in a field environment and compared with two commercial biological fungicides, Serenade^®^ (Bayer Crop Science, St Louis MO, USA) and Double Nickel^®^ (Certis Biologicals, Columbia, MO, USA), and water control. Plants were inoculated with the bacteria strains using seed treatment for early plant colonization before transplanting to a field infested with *P. capsici*. Treatments with commercial bio-fungicides followed label recommendations. Data on plant growth vigor, disease severity, number of fruits, fruit size, total yield per plant, and percent of diseased fruits displayed significant differences between the bacteria treatments. While PRT was the best treatment for most traits, followed by PSL on pepper, PSL and Double Nickel were the best treatments on tomatoes. IMC8 was best for plant vigor and larger fruit size, but with fewer fruits per plant on both crops. This study suggests bacterial isolates PRT, PSL, and IMC8 can provide additional products for growth promotion and *P. capsici* disease management in pepper and tomatoes.

## 1. Introduction

Plant diseases caused by soil-borne pathogens threaten global food security and agricultural productivity. Among these pathogens, *Phytophthora capsici* is a formidable adversary, affecting a wide range of crops, including *Capsicum annuum* (pepper) and *Solanum lycopersicum* (tomato) [1]. Though the symptoms of this disease greatly vary with the host, plant part, and environmental conditions, the symptoms on peppers and tomatoes are characterized by root and crown rot, wilting, fruit rot, and the development of dark lesions on the stems [2,3]. The pathogen spreads easily, especially in wet conditions, leading to severe economic losses in tomato and pepper crops. The disease is particularly problematic in regions with warm and humid conditions that provide favorable environments for *P. capsici* to thrive and spread rapidly [4,5,6].

The oomycete can persist in soil for extended periods, surviving as oospore-resistant structures [7]. The pathogen commonly spreads through the soil, equipment, infected plant material, and through motile zoospores in contaminated water [5,8]. In addition, *P*. *capsici* has a wide range of alternative host plant species, contributing to its persistence and prevalence in agricultural ecosystems. Substantial losses from root rot, fruit rot, and reduced fruit quality cause significant economic devastation, necessitating disease management strategies [4,7]. Traditional disease management relies heavily on chemical fungicides, but the development of resistant pathogen strains and concerns over chemical toxicities to human health, non-target organisms, and the environment need to be addressed. 

There is a growing interest in exploring sustainable and eco-friendly alternatives to chemical pesticides [9,10,11].

In nature, plants establish associations with various microorganisms and develop mutualistic interactions [12,13] including promoting plant growth by aiding the acquisition of scarce nutrients [14,15]. These microorganisms have also been shown to enhance defense mechanisms, trigger immune responses, and activate metabolic pathways that produce defense molecules, structural support, and survival compounds [11,16]. There is an intricate web of plant–microbe interactions exhibiting the potential application of beneficial microorganisms in sustainable crop production systems [17,18,19,20]. Harnessing beneficial microorganisms for improving crop production has been explored for decades, and despite great results in a controlled environment, field successes have been few [21,22,23,24,25,26,27]. In our previous research, our team isolated, characterized, and identified several bacterial endophytes that displayed efficacy against diverse fungal diseases in a controlled environment, including the Phytophthora blight of pepper and Macrophomina root rot [28,29]. This study was conducted to evaluate the effect of three bacterial endophytes in a field environment to assess their effects on plant growth promotion and protection against *P. capsici* disease.

## 2. Results

### 2.1. The Tomato Crop

**Figure 1:**
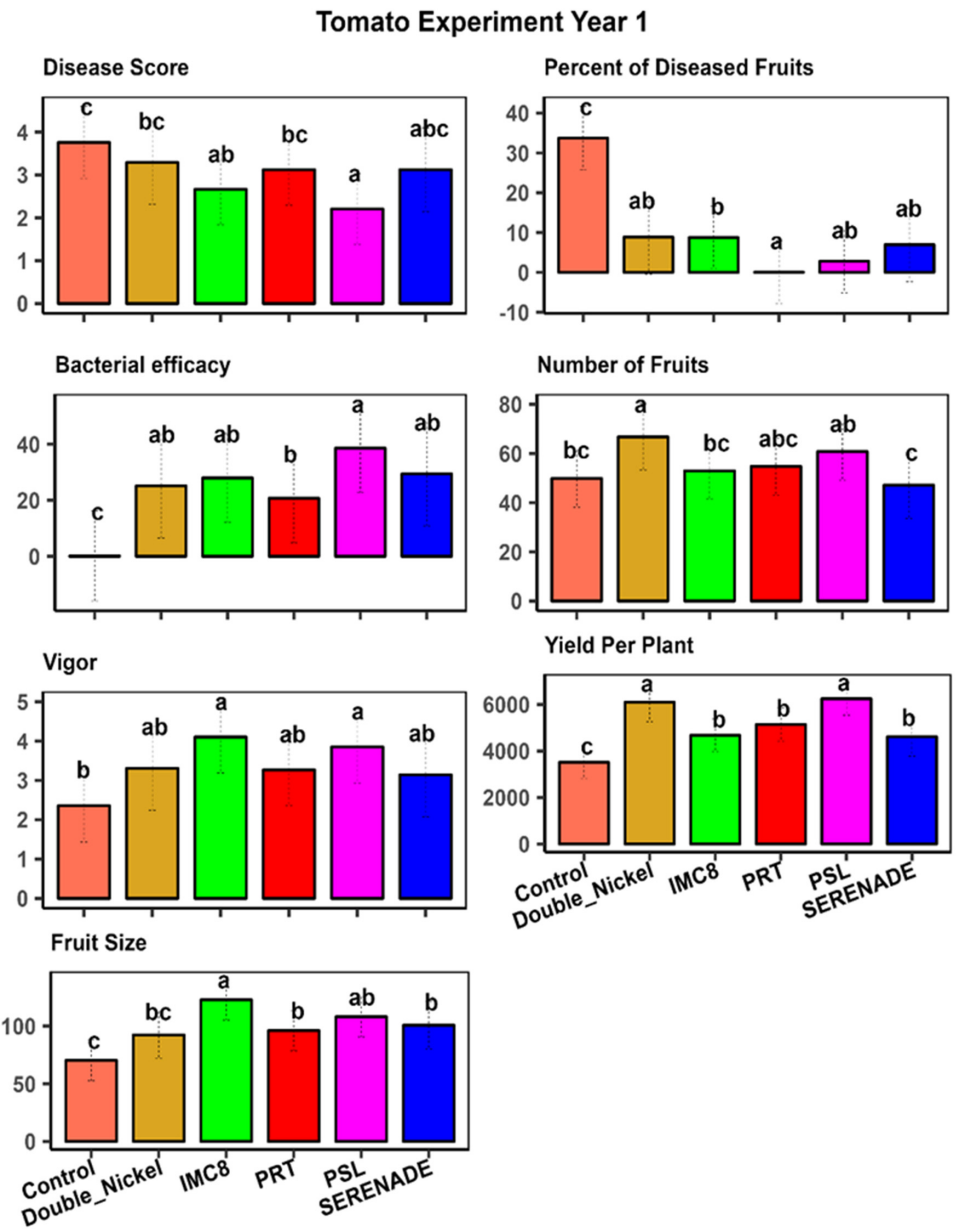
Mean separation of treatment effects for tomato traits in year 1 where visual plant size and robustness were represented by plant vigor on a scale of 1–5, with 1 being stunted growth and 5 being robust size; disease score was assessed on a 0–5 score where 0 is no disease and 5 is a severe infection and plant death. Treatments comprised bacterial isolates (PRT (*Bacillus subtilis*), PSL (*Bacillus amyloliquefaciens*), and IMC8 (*Bacillus thuringiesis*), water control, and bio-fungicides Serenade^®^ (Bayer Crop Science USA) and Double Nickel^®^ (Certis Biologials, Columbia, MO, USA). Treatments with similar letters are statistically similar at *p* ≤ 0.05.

**Figure 2:**
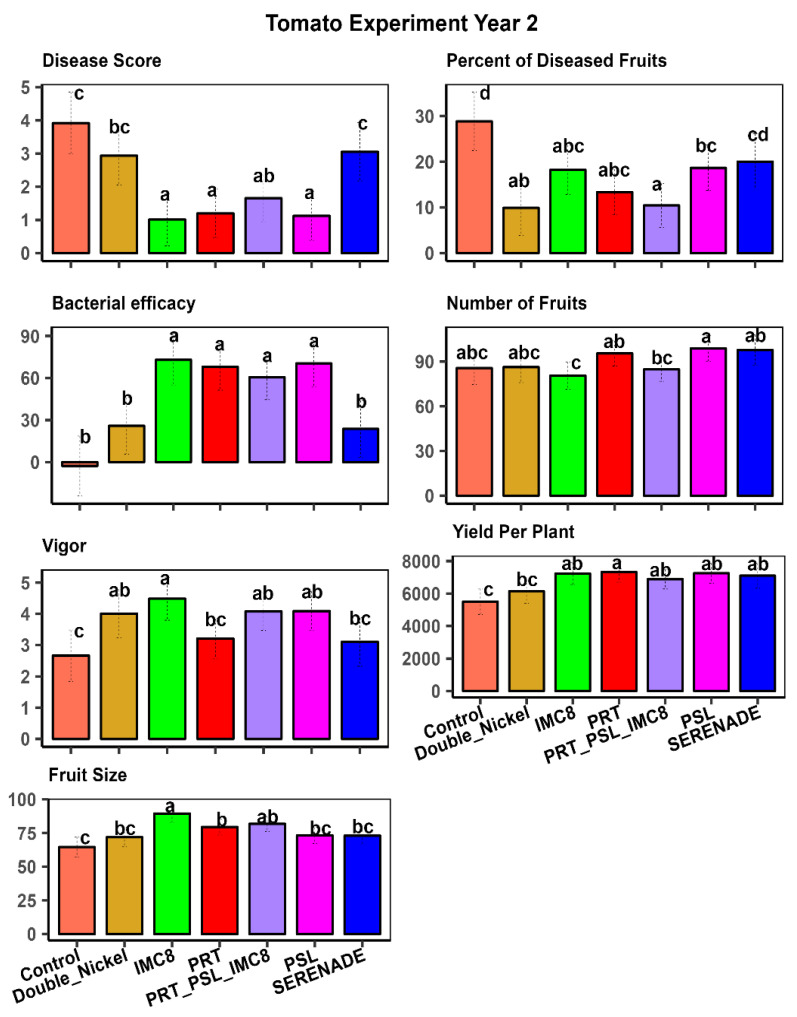
Mean separation of treatment effects for tomato traits in year 2 where visual plant size and robustness were represented by plant vigor on a scale of 1–5, with 1 being stunted growth and 5 being robust size; disease score was assessed on a 0–5 score, where 0 is no disease and 5 is a severe infection and plant death. Treatments comprised bacterial isolates (PRT (*Bacillus subtilis*), PSL (*Bacillus amyloliquefaciens*), and IMC8 (*Bacillus thuringiesis*), water control, and bio-fungicides Serenade^®^ (Bayer Crop Science US) and Double Nickel^®^ (Certis Biologials, Columbia, MO, USA. Treatments with similar letters are statistically similar at *p* ≤ 0.05.

Our study emphasizes the crucial role of Biological Control Agents (BCAs) in managing plant diseases and enhancing overall plant performance. The analysis of variance from our tomato experiments revealed significant differences between endophytic bacterial isolates in influencing key plant traits, underscoring their varying effectiveness in promoting plant health and growth, and aiding plant protection against diseases (Table 1). The subsequent mean separation analysis pinpointed the most effective BCA treatments and provided valuable insights into their impact. We observed significant interactions between year and treatment effects. Hence, we focused on the mean separation of treatments in individual year experiments. Our results on isolates PSL, IMC8, PRT, and commercially available biological fungicides Serenade^®^ (Bayer Crop Science, St Louis, MO, USA) and Double Nickel^®^ (Certis Biologicals, Columbia, MO, USA) exhibited statistically significant mean separations compared to the non-treated control. This indicated apparent differences in their capacity to manage Phytophthora root rot and improve yield. Figure 1 and Figure 2 illustrate these differences, providing a comprehensive view of the treatment comparisons. Among these treatments, PSL stands out for its consistent efficacy, effectively controlling the disease in the two-year study. In addition to its disease management capabilities, PSL consistently enhanced plant growth and health metrics, indicating its potential for robust integrated pest management (IPM) strategies. Its ability to perform reliably across multiple growing seasons highlights its value for disease control in different years.

While IMC8 effectively promoted plant growth by increasing fruit size and plant growth vigor, it also reduced the number of fruits and overall yield per plant. This suggests that while IMC8 can enhance specific growth parameters, it may not be the optimal choice for maximizing plant performance in overall yield, which is the most important parameter for farmers. The trade-off between increased fruit size and reduced yield per plant needs to be carefully considered when selecting treatments for specific agricultural goals. PRT demonstrated similar efficacy to the commercially available bio-fungicides used as the standard controls, Double Nickel^®^ and Serenade^®^, across almost all traits in the tomato experiment. This highlights PRT as a reliable alternative to these well-established treatments, offering similar benefits and alternative products in disease management and overall plant health. Its performance suggests that PRT can be effectively integrated into existing disease management programs without compromising plant health or yield (Figure 1 and Figure 2). The non-treated control plants exhibited the least growth and highest disease score, underscoring the importance of employing our isolates in disease management strategies. The significant differences observed among treatments emphasize the importance of selecting appropriate biological control agents (BCA) to optimize plant health and yield. By focusing on the mean separation of individual year experiments, our study provides an understanding of the performance of each treatment over time (Figure 1 and Figure 2).

### 2.2. The Pepper Crop

**Figure 3:**
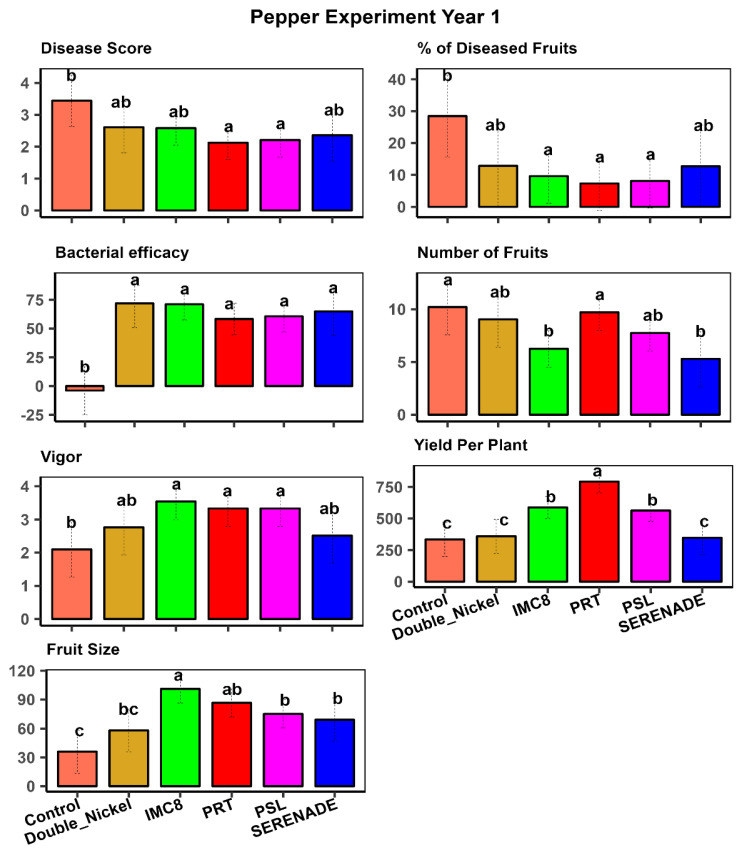
Mean separation of treatment effects for pepper traits in year 1, where visual plant size and robustness were represented by plant vigor on a scale of 1–5, with 1 being stunted growth and 5 being robust size; disease score was assessed on a 0–5 score, where 0 is no disease and 5 is a severe infection and plant death, and yield per plant was in grams. Fruit size was measured by surface diameter (mm). Treatments comprised bacterial isolates, (PRT (*Bacillus subtilis*), PSL (*Bacillus amyloliquefaciens*), and IMC8 (*Bacillus thuringiesis),* water control, and bio-fungicides Serenade^®^ (Bayer Crop Science USA) and Double Nickel^®^ (Certis Biologials, Columbia, MO, USA). Treatments with similar letters are statistically similar at *p* ≤ 0.05.

**Figure 4:**
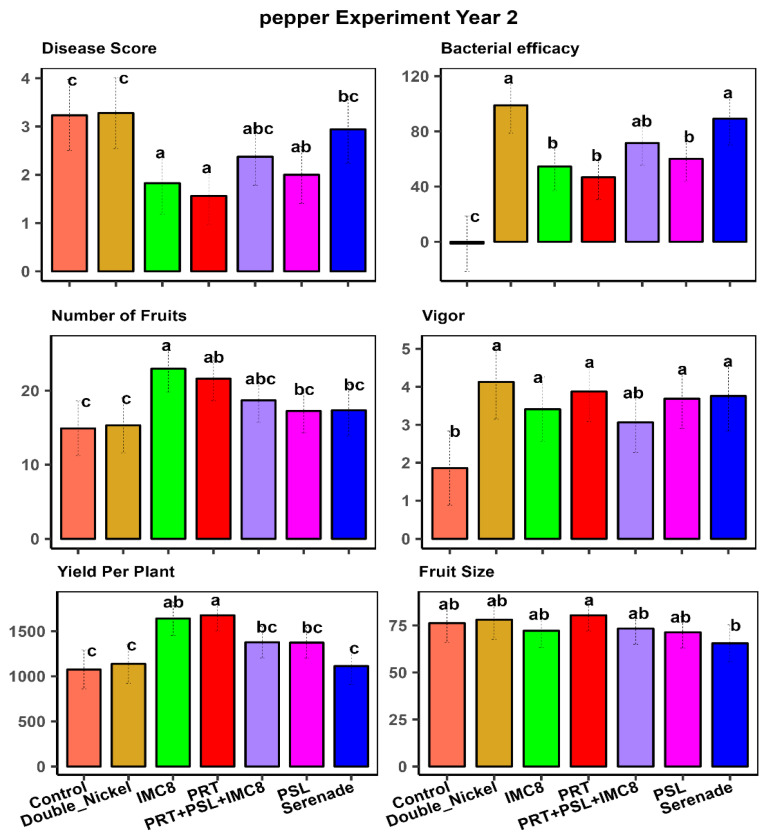
Mean separation of treatment effects for pepper traits in year 2, where visual plant size and robustness were represented by plant vigor on a scale of 1–5, with 1 being stunted growth and 5 being robust size; disease score was assessed on a 0–5 score, where 0 is no disease and 5 is a severe infection and plant death, and yield per plant was in grams and fruit size by surface diameter (mm). Bacterial efficacy was based on the number of infected fruits and the disease score. Treatments comprised bacterial isolates, (PRT (*Bacillus subtilis*), PSL (*Bacillus amyloliquefaciens*), and IMC8 (*Bacillus thuringiesis*), water control, and bio-fungicides Serenade^®^ (Bayer Crop Science USA) and Double Nickel^®^ (Certis Biologials, Columbia, MO, USA). Treatments with similar letters are statistically similar at *p* ≤ 0.05.

The analysis of variance for the pepper experiments revealed statistically significant differences between treatments for the traits measured (Table 2), just as in the tomato crop. These significant differences underscore the varying effectiveness of the treatments in managing disease and promoting plant health under field conditions. We also observed significant interactions between year and treatment effects, indicating that the effectiveness of the treatments varied across different growing seasons. Figure 3 and Figure 4 illustrate the mean separation of treatments for the first and second years of the pepper experiments. These figures provide a detailed comparison of the treatments, highlighting the differences in their effectiveness over the two years. Year-by-year analysis is crucial for understanding the consistency of each treatment over time. In contrast to the tomato experiments, where PSL was the most effective treatment, PRT emerged as the best treatment for controlling disease and promoting growth in pepper plants (Table 2, Figure 3 and Figure 4).

PRT′s superior performance in pepper underscores its potential as a key BCA for this crop. Its effectiveness in promoting growth and enhancing protection against *P. capsici* disease in pepper plants makes it a valuable tool for farmers. IMC8 consistently improved plant growth, as indicated by plant growth vigor and fruit size in pepper and tomato plants. This consistent performance across different crops highlights IMC8′s versatility and efficacy in suppressing disease severity. Its ability to enhance plant growth and increase fruit size suggests that IMC8 can be a valuable component of integrated pest management strategies for multiple crops. Additionally, in year 2, a cocktail comprising the three endophytic bacteria in a 1:1:1 ratio by volume was added to the treatments. This cocktail was equally effective as the best individual treatment in pepper and tomato experiments (Figure 2 and Figure 4).

## 3. Discussion

Phytophthora blight caused by *Phytophthora capsici* is one of the most important diseases affecting many hosts, including tomato, pepper, eggplant, cucumber, watermelon, squash, pumpkin, and melon [2,4,5,9]. *P. capsici* is especially important in the southeastern United States, where warm temperatures, high relative humidity, and frequent rainfall promote rapid disease development [2,7]. Yield losses from *P*. capsici range between 10–50%, but total crop losses from *P*. capsici root rot can occur under wet conditions [9,30]. Although host plant resistance to *P*. capsici can increase with plant age, the fruits remain susceptible throughout the growing season; stunting, girdling, or cankers may occur in less susceptible cultivars [5]. The isolates evaluated in this study have previously displayed substantial biocontrol activities against *P. capsici* and other diseases caused by diverse fungal pathogens in vitro and in vivo greenhouse environments [31,32]. However, it is well documented that the efficacy of biological agents in greenhouse-controlled environments may not translate to field environments. This study has shown that our selected BCAs were effective in field environments. As reported by other scientists, no single tool effectively controls the *P. capsici* disease. Continuous research is needed to identify the combination of tools that can reduce the impact of Phytophthora blight. Our previous greenhouse studies observed that our isolates exhibited excellent efficacy when combined with application methods, including root drenching, foliage sprays, and seed treatment [31,32]. This field study aligns with other studies that combined different procedures to improve the efficacy of biocontrol agents [33].

A combination of cultural practices, including long-term crop rotation, sanitation, field moisture management, site selection to avoid any environment conducive to the pathogen, use of variety resistance, *albeit* moderate resistance when available, and ecologically friendly products, can help reduce the use of chemical pesticides in conventional farming and provide tools for organic farming systems. Our study employed the technique of introducing the endophytes by seed treatment so as to allow early plant colonization and plant priming with the biocontrol agents before exposure to the pathogen in the field. Drenching the plants during transplanting and later during flowering is expected to reinforce the biocontrol concentration in the plants and soil and enhance the antagonistic ability of the bacteria. In this comprehensive field study, plant growth measured by vigor in size, fruit yield, and plant health measured by the percent of diseased fruits, and overall disease score per plant revealed a significant potential of these isolates in biocontrol and yield improvement for *Capsicum annuum* (pepper) and *Solanum lycopersicum* (tomato). Isolates PRT, PSL, and IMC8 displayed their consistent effects in two growing seasons. The impact of the endophytic bacteria remained evident over the two-year study, indicating a robustness in their performance despite some level of interaction with the environment. Considering the dynamic nature of field conditions and the potential challenges from varying environments, the positive effects and plant responses to the bacterial isolates further display their unique attributes. PRT emerged as the most effective treatment for most evaluated traits, particularly for pepper plants. PSL demonstrated notable efficacy on peppers and tomatoes, while Double Nickel displayed effectiveness specifically on tomatoes. IMC8, on the other hand, exhibited a distinct profile, excelling in enhancing plant vigor and fruit size but with a trade-off of a smaller number of fruits per plant, emphasizing the need for a nuanced approach based on specific crop requirements. Their consistent performance across varying environmental conditions indicates their adaptability and efficacy, which are essential for practical implementation in diverse agricultural settings.

Harnessing the naturally occurring beneficial organisms for biocontrol is essential for implementing effective integrated pest management (IPM) strategies [11,19]. The findings of this study suggest that bacterial isolates PRT and PSL hold significant promise as potential contributors to *Phytophthora capsici* IPM for yield improvement in pepper and tomatoes. The mutually beneficial relationship between the bacterial endophytes and their hosts has been reported to include plant growth promotion, fixing atmospheric nitrogen, increasing mineral nutrient uptake, inducing plant defense mechanisms, and reducing disease severity [11,12,13,14,28,34,35]. Preliminary results on the isolates used in this study have indicated the production of growth hormones, surfactants, and various enzymes and biochemical compounds associated with plant nutrition and defense against pathogens [28].

## 4. Materials and Methods

### 4.1. Description of the Experimental Site

The study was conducted at the Tennessee State University Agricultural Research and Extension Center (AREC) in Nashville, situated at 36°10′36′ N and 86°49′34′′ W, with an altitude of 128 m above sea level. The research station is in the floodplain of the Cumberland River. The experimental site′s soil is classified as silt loam, belonging to the Byler series [29]. The climatic conditions are characterized by a humid subtropical climate (Köppen classification: Cfa), with an annual rainfall of approximately 1200 mm and temperatures ranging from 30 °C to 32 °C during summer months, with occasional extreme temperatures reaching 40 °C during the day and 30 °C at night. The field study occurred from May to September, aligning with Tennessee′s planting season for peppers and tomatoes.

### 4.2. Treatment and Experimental Design

The study evaluated the efficacy of three endophytic bacterial isolates individually and in a 1:1:1 (*v*:*v*) cocktail of the isolates against *Phytophthora capsici* and compared them with two commercial bio-fungicides, Double Nickel and Serenade, and water control. Separate experiments were carried out on tomatoes ‘Rutgers’ and sweet peppers ‘California Wonder’, both susceptible to *P. capsici*. The cocktail treatment was introduced in the second year. The seeds were soaked in water for 12 h at 4 °C and then blotted dry before soaking in a bacterial suspension of 1 × 10^8^ colony-forming units (cfu)/mL for one hour; the control treatment was water. The treated seeds were then planted in Miracle-Gro^®^ potting mix, and after two months they were transplanted into field soil infested with *P. capsici*. The field plan consisted of a completely randomized block design using a replication of four plots. Each plot contained five seedlings, spaced 1 m apart in rows. Plastic mulching and drip irrigation were employed for weed control, and drip irrigation was provided as needed. No fertilizer was used throughout the experiment over two years. At the time of transplanting, each seedling was drenched with 50 mL of bacterial suspension of 1 × 10^8^ cfu/mL. During early flowering and fruiting stages, plants were resprayed to run off using a bacterial suspension of 1 × 10^8^ cfu/mL.

### 4.3. Pathogens Inoculum Preparation and Culture Conditions

The *P*. *capsici* pathogen was previously isolated from soil in the experimental area using PARP (pentachloronitrobenzene, ampicillin, rifampicin, and pimaricin)-amended V8 juice agar media following the protocol outlined in Jeffers [30]. *The P. capsici* isolates were maintained on clarified V8^®^ juice agar media consisting of 800 mL distilled water, 200 mL clarified V8 juice, 2 g CaCO_3_, and 15 g bacteriological agar. The pathogen was cultured on V8 agar media for seven days and flooded with water for 36 h under fluorescent light to facilitate zoospore production for greenhouse experiments to confirm the pathogen virulence in [27,32]. Pepper and tomato were grown in the area for two years, and a high infestation of *P. capsici* in the experimental area was confirmed.

### 4.4. Bacterial Endophytes

Three promising bacterial endophytes, namely *Bacillus subtilis* (PRT), *Bacillus thuringiensis* (IMC8), and *Bacillus amyloliquefaciens* (PSL), were selected based on previous studies [28,31,32]. These endophytic bacterial isolates were originally isolated from different plants and tissues and were chosen based on their potential biocontrol activity against *P. capsici,* as indicated by the previous studies conducted by [31,32]. The bacterial isolates were cultured and maintained following established protocols outlined in Jeffers [32].

### 4.5. Plant Trait Measurements

Data on various plant traits were recorded, including plant vigor assessed by the visual appearance of plant size and robustness on a scale of 1–5, where 1 is stunted growth and 5 is robust size, disease score on a 0–5 score, where 0 is no disease and 5 is a severe infection causing plant death [32], number of fruits (NF), fruit size (FS), and total yield per plant (TYLD). Additionally, secondary data, such as the percentage of diseased fruits (PDF), was calculated using the formula:

$$\left(\frac{Number\;of\;diseased\;fruit\;score}{Number\;of\;Fruit}\right)\times \;100$$

Endophytic Bacterial Efficacy (EBF) was measured by using the formula:

$$1\;-\;Number\;of\;fruits\left(\frac{Treatment\;disease\;score}{Control\;disease\;score}\right)\times \;100$$

### 4.6. Statistical Analysis

A two-step analysis of variance (ANOVA) was conducted using a mixed linear model. The first ANOVA was performed on individual crop data for each year, followed by a combined year data analysis for each crop. The mixed linear model considered treatments as fixed effects, while individual plants, blocking, and year were treated as random effects. The statistical analysis was carried out using R statistical software. Analysis of variance was conducted using the “lme4” package [36], mean separation on significant differences was conducted using “means” [37] and “multcomp” [38] packages, finally, visualization was done using “ggplot2” [39], “ggpubr” [40], and [41] packages in R.

## 5. Conclusions

This research contributes valuable insights into sustainable agriculture by highlighting the potential of endophytic bacterial strains as biocontrol agents in the field environment. Future studies could delve deeper into the underlying interactions between the introduced and the native microorganisms to enhance the microbes′ beneficial effects on plants at different stages of plant colonization. The observed differences in efficacy among the isolates presented in this study provide a foundation for further exploration and optimization of these microbial interventions in farmers′ fields. The study demonstrates the potential of harnessing natural products that colonize plants as endophytes as a viable disease control and yield improvement strategy. As agriculture faces increasing challenges, exploring eco-friendly alternatives becomes imperative, and the findings of this study contribute to the growing body of knowledge aimed at reducing the usage of chemical pesticides by using eco-friendly microbes and promoting sustainable and effective agricultural production practices.

## Figures and Tables

**Table 1:** Mean squares of individual and combined data for different traits in two years of tomato crop.

Year 1							
Sources of Variance (df)	Disease Rating	Plant Vigor	Number of Fruits	Diseased Fruits	Yield Per Plant (g)	Fruit Size	Bacteria Efficacy
Treatment (5)	4.86 ***	5.91 ***	707.88 **	1914 ***	15,945,932 ***	5108.10 ***	14,302.2 ***
Block (3)	0.89	1.06	74.45	91	719,588	575.00	537.2
Plant (3)	1.02	0.52	35.48	47	247,442	92.70	393.9
Residuals (96)	0.86	1.04	165	78	625,899	379.00	510.5
**Year 2**							
Treatment (6)	12.49 ***	4.84 ***	637.28 ***	461 ***	4,794,532 ***	633.22 ***	7182.7 ***
Block (3)	1.73	0.05	468.13 *	109	2,447,758 *	215.76 *	1405.7
Plant (3)	0.72	0.08	54.31	69	236,631	8.90	262.3
Residuals (71)	0.96	0.73	130.49	45	704,153	63.14	596.2
**Combined Analysis**							
Year (1)	64.53 ***	5.81 *	52,671 *	1930 ***	136,860,039 ***	24,973.20 ***	36,694 ***
Treatment (6)	13.45 ***	9.52 ***	1282 ***	1695 ***	14,751,416 ***	4959.90 ***	16,666 ***
Block (3)	1.18	0.60	96	43	1,890,524	161.10	916
Plant (4)	0.85	0.35	10	89	244,906	64.30	224
Treatment: Year	4.09 ***	0.53	701 ***	380.45 ***	6,427,436 ***	910.6 ***	2963 ***
Residuals (177)	0.92	0.88	170	73	828,298	267.50	

NB: Degree of freedom in brackets; *** significant at *p* ≤ 0.001, ** significant at *p* ≤ 0.01, and * *p* ≤ 0.05. Plant vigor was based on visual plant size and robustness on a scale of 1–5, where 1 is stunted growth and 5 is robust size. Disease rating score was on a 0–5 scale, where 0 is no disease and 5 is a severe infection and plant death; fruit size by surface circumference and bacterial efficacy based on a combination of disease score and the number of infected fruits.

**Table 2:** Mean squares of individual and combined data for different traits in two years of pepper crop.

Year 1							
Sources of Variance (df)	Disease	Vigor	Number of Fruits	% Diseased Fruits	Yield/Plant	Fruit Size	Bacteria Efficacy
Treatment (5)	4.08 **	5.89 ***	60.57 ***	1110.01 ***	571,764 ***	7908.7 ***	10,703.4 ***
Block (3)	1.23	1.14	15.14	77.93	109,329	791.8	755
Plant (5)	1.05	1.22	12.34	479.68	15140	1556.1	1467.7
Residuals (96)	0.93	0.98	9.93	237.79	25638	718.7	739.1
Year 2							
Treatment (6)	6.5 ***	6.67 ***	108.85 ***	119.91	759,462 ***	359.35 *	12,132.5 ***
Block (3)	1.93	1.75	16.84	86.95	60,439	271.71	1254.7
Plant (3)	0.07	1.12	20.9	28.59	25,983	360.76	92.7
Residuals (83)	0.74	1.31	18.78	124.27	63,544	146.39	554
Combined analysis							
Year (1)	4.2 *	5.80 *	4561.3 ***	1042.33 *	29,804,095 ***	47.5	353.3
Treatment (7)	7.15 ***	8.49 ***	249.3 ***	342.81	1,673,176***	3532.5 ***	16,627.7 ***
Block (3)	0.78	1.44	4561.3	116.82	60,163	989.3	31
plant (5)	2.96	1.47	9.6	334.28	15,058	516.5	298.9
Treatment: Year (4)	1.74	2.78	149.5 ***	827.52 ***	104,206	4388.6 ***	1686.5 *
Residuals (183)	0.87	1.14	14.1	185.67	44,214	471.5	617.3

NB: df = Degree of freedom in brackets; *** Significant at *p* ≤ 0.001, ** Significant at *p* ≤ 0.01, * Significant at *p* ≤ 0.05 where Plant vigor is visual plant size and robustness on a scale of 1–5, where 1 is stunted growth and 5 is robust size, disease rating score assessed on a 0–5 score, where 0 is no disease and 5 is a severe infection and plant death.

## Data Availability

The original contributions presented in this study are included in the article. Further inquiries can be directed to the corresponding author.
